# Galectin-9 in Gastroenterological Cancer

**DOI:** 10.3390/ijms24076174

**Published:** 2023-03-24

**Authors:** Asahiro Morishita, Kyoko Oura, Tomoko Tadokoro, Tingting Shi, Koji Fujita, Joji Tani, Masanori Atsukawa, Tsutomu Masaki

**Affiliations:** 1 Department of Gastroenterology and Neurology, Kagawa University Faculty of Medicine, Miki 761-0793, Japan; 2 Department of Gastroenterology and Hepatology, Nippon Medical University Hospital, Tokyo 113-8603, Japan

**Keywords:** galectin-9, gastroenterological cancer, apoptosis, immunotherapy

## Abstract

Immunochemotherapy has become popular in recent years. The detailed mechanisms of cancer immunity are being elucidated, and new developments are expected in the future. Apoptosis allows tissues to maintain their form, quantity, and function by eliminating excess or abnormal cells. When apoptosis is inhibited, the balance between cell division and death is disrupted and tissue homeostasis is impaired. This leads to dysfunction and the accumulation of genetically abnormal cells, which can contribute to carcinogenesis. Lectins are neither enzymes nor antibodies but proteins that bind sugar chains. Among soluble endogenous lectins, galectins interact with cell surface sugar chains outside the cell to regulate signal transduction and cell growth. On the other hand, intracellular lectins are present at the plasma membrane and regulate signal transduction by regulating receptor–ligand interactions. Galectin-9 expressed on the surface of thymocytes induces apoptosis of T lymphocytes and plays an essential role in immune self-tolerance by negative selection in the thymus. Furthermore, the administration of extracellular galectin-9 induces apoptosis of human cancer and immunodeficient cells. However, the detailed pharmacokinetics of galectin-9 in vivo have not been elucidated. In addition, the cell surface receptors involved in galectin-9-induced apoptosis of cancer cells have not been identified, and the intracellular pathways involved in apoptosis have not been fully investigated. We have previously reported that galectin-9 induces apoptosis in various gastrointestinal cancers and suppresses tumor growth. However, the mechanism of galectin-9 and apoptosis induction in gastrointestinal cancers and the detailed mechanisms involved in tumor growth inhibition remain unknown. In this article, we review the effects of galectin-9 on gastrointestinal cancers and its mechanisms.

## 1. Introduction

Apoptosis, a type of programmed cell death other than necrosis, is morphologically crucial for the development and maintenance of tissues. Tissues require apoptosis to maintain normal morphology, quantity, and functionality [1]. When apoptosis is triggered, cell membranes are maintained stable, and the release of inflammation-inducing substances such as cytokines from the destroyed cells is prevented, thus preventing inflammation and tissue injury [2]. Failure of apoptosis results in an imbalance between cell division and death, which disrupts tissue homeostasis and leads to the accumulation of dysfunctional cells, an excess of which is thought to contribute to carcinogenesis [3].

Lectins, “glycan-binding proteins that are neither enzymes nor antibodies”, are found in a wide variety of tissues and can functionally influence cell fate [4]. Among soluble endogenous lectins, galactoside-binding lectins, or galectins, are thought to interact extracellularly with cell surface glycans, regulate signal transduction, and determine cell fate [5,6,7,8]. Among the various animal lectins, galectin-9, encoded by the LGALS9 gene, is a 36-kDa protein and a β-D-galactoside mammalian lectin [9,10,11,12,13]. It was identified in murine embryonic kidneys and human Hodgkin’s lymphoma tissues [10,11]. Several recent reports have demonstrated that galectin-9 is expressed in various tumors, including those in the liver, small intestine, thymus, kidney, spleen, lung, heart, and skeletal muscles [14]. Galectin-9 has been shown to be expressed on the surface of thymocytes, induce apoptosis of T lymphocytes, and play an essential role in immune self-tolerance through negative selection in the thymus [11]. Furthermore, treatment with galectin-9 induces apoptosis in human cancer and immunocompetent cells, such as T lymphocytes. In other words, galectin-9 has been suggested to be a candidate for anticancer drugs based on its glycan recognition function [15,16]. We have demonstrated the effect of galectin-9 in various gastroenterological cancers, such as esophageal cancer [17], hepatocellular carcinoma [18], cholangiocarcinoma [19], and pancreatic cancer [20]. However, little is known about the relationship between galectin-9 and apoptosis in gastroenterological cancers.

Here, we review apoptosis and galectin-9 in gastrointestinal cancers and assess the potential of galectin-9 as an anticancer agent for various gastrointestinal cancers.

## 2. Apoptosis and Malignancy

### 2.1. Apoptosis, Programmed Cell Death

Apoptosis is a proposed type of programmed cell death [1] characterized by morphological changes occurring in response to extrinsic and intrinsic stress [21]. Apoptosis is also induced during normal tissue development and morphogenesis [22]. Once apoptosis is initiated, cell membranes remain stable, thus preventing the release of cytokines and other pro-inflammatory substances from destroyed cells and attenuating inflammation and tissue injury [2]. 

The caspase and B-cell lymphoma 2 (BCL-2) protein families play crucial roles in executing or halting apoptosis. Caspases are cysteine-dependent, aspartate-specific peptidases that initiate or affect apoptotic and inflammatory pathways. [23]. The BCL-2 protein family is a key modulator of apoptosis, and its members share Bcl-2 homology (BH) domains (BH1-4) [24].

Apoptosis has extrinsic and intrinsic subtypes depending on the mechanism of the signaling pathway. The extrinsic apoptotic mechanism operates by binding ligands to cell surfaces death receptors, such as tumor necrosis factor (TNF) and TNF Receptor-1, Fas Ligand and Fas Receptor, and TNF-related apoptosis-inducing ligand (TRAIL) and TRAIL Receptors-1 and -2 [25]. Death receptors share intracellular death domains, and their stimulation causes death domains to form oligomers with the death receptor, thus changing its conformation. The Fas-associated protein with the death domain and the TNF Receptor-1 associated death domain protein, which are death receptors and adaptor proteins, then activate the apoptotic initiator procaspase 8/10 [26]. The intrinsic apoptosis signaling pathway is characterized by mitochondrial changes in response to various stress signals while activating the initiator procaspase-9 [27]. Mitochondrial pro-apoptotic BH3-only proteins antagonize anti-apoptotic proteins, such as B-cell lymphoma 2 (BCL-2), Bcl-xL B-cell lymphoma-extra large (BCL-xL), B-cell lymphoma-w (Bcl-w), and myeloid cell leukemia-1 (MCL-1). When the mitochondrial outer membrane disrupts, cytochrome c leaks into the cytoplasm [28]. Cytochrome-c and Apaf-1 in the cytoplasm form a complex called the apoptosome, which coexists with the initiator procaspase-9. External and internal apoptosis share the execution factor caspase-3/6/7, which cleaves critical cellular components and induces cell death [29]. The role of the extrinsic apoptotic cascade is to recruit the intrinsic pathway via BH3-interacting domain death agonist (BID), a BH3-only protein, when the cell is not killed by death receptor–ligand interactions alone [3]. Caspase-8 is then activated and cleaves Bid to generate tBid fragments, resulting in mitochondrial outer membrane permeability in the intrinsic pathway [30]. 

Via the various pathways described above, apoptosis disposes of damaged or abnormal cells, especially cancer cells, in a manner that is not harmful to the organism.

### 2.2. Cancer and Apoptosis

Tumor cells have evolved several abilities to evade apoptosis [31,32,33,34,35]. The most common adaption is the loss of tumor suppressor function, which eliminates this important damage sensor from the apoptosis-inducing circuitry [32]. Tumors may achieve similar results by increasing the expression of anti-apoptotic regulators and survival signals, suppressing pro-apoptotic factors, and short-circuiting exogenous ligand-induced death pathways. The diversity of apoptosis evasion mechanisms may reflect the diversity of apoptosis-inducing signals encountered during the malignant transformation of cancer cell populations. Evasion of apoptosis is one of the most important features of cancer, along with the ability to replicate endlessly, sustain angiogenesis, invade tissues, and induce metastasis [34]. Apoptosis evasion allows adequate elimination of DNA-damaged cells and prevents abnormal cell proliferation [1,36,37]. Furthermore, dysregulation of apoptosis is deeply involved in the resistance of cancer cells to chemotherapy [33,34,38]. To date, various drugs have been investigated and developed to target molecules related to apoptosis at the RNA and protein levels. However, all of these drugs have been inadequate and repeatedly lost their effectiveness, as cancer eventually became resistant to the drugs. Therefore, it is critical to lubricate and regulate apoptosis as much as possible by glycan–protein binding and various glycan-mediated actions, which leads to the suppression of cancer cell mitosis. Since glycans can covalently bind to at least nine different amino acids [39,40], proteomic information is regulated and diversified by exponentially differentiating glycan structures or the glycome. The structure of apoptotic mechanisms and programs, as well as the strategies used by cancer cells to evade their effects, were widely understood as early as a decade ago. Since then, the most notable conceptual advances have involved other forms of cell death that broaden the scope of “programmed cell death” as a barrier to cancer. Galectins have a strong influence on tumor progression by modulating cell proliferation, evasion of growth inhibitors and immune responses, resistance to cell death, induction of angiogenesis, invasion, and metastasis. 

Therefore, the binding of glycans to proteins and the actions mediated by various glycans, which do not belong to the conventional mechanism causing drug resistance, may be new targets for drugs that control apoptosis and, ultimately, inhibit cancer cell proliferation.

### 2.3. Apoptotic Signaling Is Modulated by Glycosylation of Proteins 

Cell surface receptor proteins associated with apoptosis are modified by glycans, which alter protein structure, conformation, and function [39]. Glycan structures are diversified by the association of ten types of monosaccharides with different components to form glycans [40]. The binding of glycans to proteins also generates various glycan complexes [41,42]. Therefore, information on proteomics is controlled and diversified by exponentially differentiating glycan structures or the glycome. Unlike proteins, glycosylation does not use DNA as a template and is, therefore, influenced by factors such as the presence or absence of glycan substrates and the activity and expression levels of enzymes and transporters [43]. As an exogenous apoptotic pathway, glycosylation is stabilized by two N-glycosylation sites on the Fas ligand [44] and contributes to exogenous apoptosis initiation via DISC–DISC interaction and procaspase 8 oligomerization. The Fas ligand and TRAIL receptors, which activate apoptosis upon binding, also depend on glycosylation for their proper functioning. In addition, excessive glycosylation of the Fas ligand renders Fas unable to oligomerize, and apoptosis is not induced [45]. TRAIL Receptors-1 and -2 have also two potential O-glycosylation sites, and fucosylation of the core 2 O-glycan enhances the sensitivity of tumor cells to TRAIL-induced apoptosis [46,47]. Therefore, glycosylation of molecules crucial for apoptosis initiation may modulate apoptosis by regulating the affinity of the receptor the ligand.

### 2.4. Lectins Regulate Diverse Signaling through Glycosylation

Glycan-binding lectins, which are glycan ligands, are important molecules regulating the apoptotic pathway [48]. Lectins bind to glycans and cause interaction with glycosylated death receptors, altering the affinity of pro-apoptotic molecules for their ligands [49,50,51]. Lectins on the cell membrane form a lattice structure by binding to and bridging between sugar chains. This is thought to anchor receptors on the cell surface and stop their rotation [52]. A galectin family is a group of galactoside-specific lectins with highly conserved carbohydrate recognition sites that have been shown to cause negative selection of T lymphocytes by apoptosis in the thymus and contribute to acquired immunity [11]. As for endogenous galectins, they are thought to play an important role in tumor control. [53]. 

Thus, galectins are thought to regulate apoptosis-related signaling pathways and control tumor growth by modulating ligand–receptor affinity.

## 3. Galectin-9

### 3.1. Structure of Galectin-9

Galectin-9, with a molecular weight of 36 kDa, a β-D-galactoside mammalian lectin, is encoded by the LGALS9 gene [9]. It was identified in murine embryonic kidneys and human Hodgkin’s lymphoma tissues in 1997 [10,11]. Galectin-9 has a specific affinity to the lactosyl group and two different but homologous carbohydrate recognition domains in the N-terminus and C-terminus. The two domains are connected by linker peptides, which are tandem repeat-type peptides; the lengths of these linker peptides vary among galectins and exhibit three isoforms. Identification and characterization of galectin-9 have been conducted since 1991. ConA-activated CD4+ T lymphocytes secrete ecalectin, an eosinophil chemoattractant with a molecular weight of 30–40 kDa [45,46]. Ecalectin was first recognized as a variant of galectin-9 that was later found to be the medium-sized galectin-9 isoform [14,47]. In addition, the urate transporter was also identified as galectin-9, an especially long isoform of galectin-9 in humans and mice [13,48]. Several isoforms are included in the galectin-9 protein, which is transcribed from LGALS9. The LGALS9 gene is encoded on the short arm of chromosome 17q11.2 (HGNC: 6570) and consists of 355 amino acid peptides after translation [9]. Additionally, 17q11.2 also encodes two transcripts, LGALS9B (HGNC:24842) and LGALS9C (HGNC:33874), which have nearly identical sequences, suggesting a possible gene duplication. The details of the expressions and functions of these two galectin-9-like molecules are not well understood.

Galectin-9 is composed of two β-galactoside binding sites joined by a peptide [11]; the C-terminal carbohydrate recognition domain (C-CRD), like the N-terminal carbohydrate recognition domain (N-CRD), is composed of two inversely parallel S1–S6 β sheets and an F1–F5 β strand and α helix [54]. The glycan-binding pockets of the strands differ from each other in amino acid sequence and thus have different affinities and biological activities toward β-galactosides [55,56]. However, both N-CRD and C-CRD are required for the chemoattraction of eosinophils by galectin-9 [57]. Galectin-9 has a protease-sensitive site in the linker peptide and, like the galectin prototype, is degraded into two monovalent sugar-specific proteins [58]. Fifty-eight amino acid linker peptides represent long-chain isoforms (galectin-9L or galectin-9FL). Twenty-six and fourteen amino acid linker peptides represent medium- (galectin-9M) and short- (galectin-9S) chain galectin-9 [59]. These linker peptides are not critical for some CRD-dependent physiological effects [13,58,59]. However, the length of the linker peptide is involved in the structure of galectin-9-glycan oligomerization and lattice formation at the cell surface [60]. Deletion of the transcript of exon 10 from the messenger RNA of galectin-9 results in the truncation of C-CRD encoded in exon 11 [7], and the truncation of C-CRD directly influences the glycan specificity for each galectin-9 isoform. The functional diversity of galectin-9 isoforms due to the length of the linker peptide and C-CRD truncation remains to be clarified.

Thus, the structure and diversity of galectin-9 should be elucidated in future studies to determine how it is involved in cancer and apoptosis.

### 3.2. Secretion of Galectin-9

Galectins, including galectin-9, cannot be secreted extracellularly via the conventional protein secretory pathway due to the lack of hydrophobic signal peptides [61,62]. Galectin-9 has recently been shown to be released extracellularly via a non-classical pathway involving exosomes. For example, when galectin-9 is expressed on the cell surface in the Jurkat T cell line and T cells are stimulated with this galectin-9 antigen, the secretion of exogenous galectin-9 isoforms from T cells increases through a non-classical pathway [62]. Mouse CD4+ T cells extruding galectin-9 to the cell surface also secreted soluble galectin-9, and the amount secreted increased upon T-cell receptor stimulation [63]. In nasopharyngeal carcinoma cells, infection with the Epstein–Barr virus resulted in the release of galectin-9-containing exosomes into the culture medium [64,65]. These results indicate that galectin-9 is released extracellularly via a non-classical pathway involving galectin-9-containing exosomes and that galectin-9 in exosomes is transported to and regulated by organs throughout the body.

### 3.3. Distribution of Galectin-9

**Figure 1:**
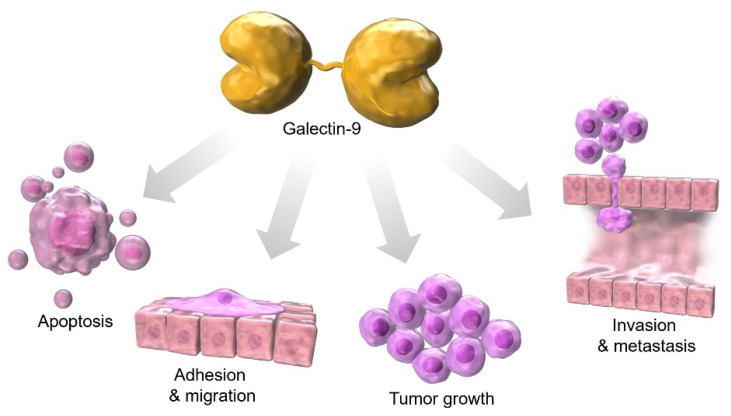
Galectin-9 is associated with apoptosis, adhesion, migration, tumor growth, invasion, and metastasis during cancer development.

Galectin-9 expression is distributed in various tumor organs, including the liver, small intestine, thymus, kidney, spleen, lung, heart, skeletal muscle [14], brain [66], placenta, pancreas, prostate, and colon [67]. Among them, predominantly expressing cells include leukocytes [68,69,70], which are responsible for innate and acquired immunity [71], thymocytes [14], activated endothelial cells [72,73], and fibroblasts stimulated by interferons [74]. Among malignant tissues, galectin-9 expression on the cell surface has been reported to be reduced in hepatocellular carcinoma (HCC) as well as in other solid tumors, such as prostate cancer [75], cervical cancer [76], and skin cancer [77,78]. Galectin-9 expression is enhanced in oral cancer [79], pancreatic cancer [80], and hematologic malignancies [10] compared to normal adjacent tissues. The galectin-9-glycan lattice has high diversity based on the complex carbohydrate that consists of ten different monosaccharides, the different affinity of the two glycan recognition domains (CRDs) of galectin-9, and the freedom of CRD rotation by flexible linker peptides, which can be used to organize the cell membrane domain and determine cell signaling. Galectin-9 has the potential to play three major roles in cell biology: (1) organizing cell membrane domains, (2) determining cell signaling thresholds, and (3) limiting the residence time of receptors at the cell surface. These functions of galectin-9 are involved in apoptosis, adhesion, migration, tumor growth, invasion, and metastasis during cancer development [8] (Figure 1). In this article, we discuss the relevance of galectin-9 in gastrointestinal cancers.

### 3.4. Galectin-9 and Its Ligands and Clinical Trials

Four clinical trials (updated to February 2023) have been registered by the United States National Institutes of Health (https://clinicaltrials.gov/, accessed on 24 February 2023) for gastroenterological cancer, and two trials have been completed. However, many previous galectin-9-related trials against solid tumors have neither been withdrawn nor terminated [81], suggesting that some galectin inhibitors are not effective and that efficacy might also depend on protocol design and galectin expression profiles in each individual.

In recent years, immunotherapy has been used to treat a variety of cancers. Recently, immunotherapy using monoclonal antibodies that inhibit immune checkpoint molecules has shown promising progress. However, such antibodies are often ineffective, and we have begun to combine them with galectin inhibitors to enhance therapeutic efficacy. Clinical trials using the galectin-3 inhibitor (DG-MD-02) in combination with ipilimumab (anti-CTLA-4) or pembrolizumab (anti-PD-1) in patients diagnosed with melanoma, non-small cell lung cancer, or squamous cell head and neck cancer have recently been conducted. In one of these trials, pembrolizumab and GR-MD-02 demonstrated promising initial results in the treatment of patients with advanced melanoma. Although the trial showed positive results, the detailed mechanisms are unknown, and further clinical trials are needed to demonstrate the efficacy of this approach and evaluate possible side effects. Clinically effective anti-PD-1 and anti-CTLA-4 antibodies, as well as TIM-3, a galectin-9 binding partner and a negative regulator of T cells, have been used as novel targets in tumor immunotherapy to enhance T cell antitumor function [82,83]. Although these clinical trials are still in their early stages, they are expected to achieve antitumor effects by blocking multiple negative regulators on T cells.

## 4. Esophageal Cancer and Galectin-9 Apoptosis 

Esophageal cancer ranks seventh as the most common cancer worldwide in terms of incidence, and it is the sixth leading cause of mortality from cancer in 2020. Approximately 70% of cases occur in males, especially in eastern Asia [84]. The two major histologic subtypes are esophageal squamous cell carcinoma (ESCC) and esophageal adenocarcinoma (EAC). The prognosis of esophageal cancer depends on the tumor stage at diagnosis; endoscopic resection methods, including endoscopic mucosal resection (EMR) and endoscopic submucosal dissection (ESD), showed benefits for early-stage patients [85,86]. Advanced stages have a poor prognosis, although surgical techniques have been advanced and optimized. Chemotherapy, radiotherapy, chemoradiotherapy, and immunotherapy may improve the outcomes of advanced-stage patients [86]. New anticancer agents and novel therapeutic strategies are crucial for improving the prognosis.

A clinical study suggested that high levels of TIM-3 and low levels of galectin-9 are associated with a poor prognosis for ESCC patients, although TIM-3 and galectin-9 are not identified as independent indicators [87]. In a previous report, the expression of galectin-9 and TIM-3 was evaluated using immunohistochemistry in human esophageal cancer tissues. Therefore, it is difficult to distinguish between extracellular and intracellular galectin-9. The functions of extracellular galectin-9 (induction of apoptosis in inflammatory cells) and intracellular galectin-9 (induction of apoptosis in cancer cells) differ. In addition, TIM-3-positive cells may reduce due to exogenous galectin-9-induced apoptosis. Our previous studies indicated that galectin-9 induces mitochondria-mediated apoptosis of ESCC cells and suppresses cell proliferation through JNK and p38 activation. Moreover, galectin-9 upregulated the expression of miR-222-5p, miR-582-5p, miR-6131, and downregulated the expression of miR-4639-5p [88]. In EAC cells, galectin-9 inhibits cell proliferation by inducing apoptosis and suppressing autophagy, which increases the levels of miR-6131 and interleukin-8 (IL-8) [17]. Galectin-9 inhibits ESCC and EAC cell proliferation mainly through inducing apoptosis, while heat shock protein 60 (Hsp60) and IL-8 axis may promote apoptosis resistance in cancers [89]. Moreover, a large-scale retrospective analysis showed that high serum IL-8 levels and tumor neutrophil infiltration are associated with worse prognoses in advanced cancers (Table 1). In addition, patients with elevated baseline serum IL-8 levels are less likely to benefit from the immune–checkpoint inhibitors nivolumab and/or ipilimumab [90]. Thus, an increase in IL-8 may be a reason for galectin-9 resistance. Endogenous galectin-9 expression in cancers has been associated with cancer cell adhesion or metastasis. The high levels of endogenous galectin-9 increase the cellular adherence ratio to promote cancer cell aggregation, whereas reduced endogenous galectin-9 expression is related to cancer cell invasion and metastasis [81,91]. Additionally, the expression of galectin-9 and interactions with its ligands regulate immune responses. Previous studies suggested that the TIM-3-galectin-9 pathway can induce T-cell apoptosis and protect cancer cells against cytotoxic T-cell-induced death, which promotes immune escape [92]. Galectin-9 expression also regulates macrophage M1/M2 polarization [93], while tumor-associated macrophages are generally M2-like and facilitate tumor growth via induction of immune suppression in various tumors [94]. With the development of immunotherapy, galectin-9 has emerged as a target to regulate tumor immune responses [95].

## 5. Gastric Cancer and Galectin-9 Apoptosis

The incidence of gastric cancer has uniformly decreased [116], and the prognosis of early gastric cancer has improved with the establishment of treatment methods such as endoscopic submucosal dissection [117]. However, gastric cancer remains one of the leading causes of cancer-related deaths worldwide [118], and unresectable advanced gastric cancer is still considered to have a poor prognosis. Systemic chemotherapy, radiation therapy, surgery, immunotherapy, and targeted therapy have proven effective against gastric adenocarcinoma, and multidisciplinary therapy is the most important treatment option [119].

In recent years, classification based on molecular subtypes of gastric cancer has facilitated personalized treatment. Investigations of biomarkers, particularly microsatellite instability (MSI), programmed cell death ligand 1 (PD-L1), human epidermal growth factor receptor 2 (HER2), tumor mutation levels, and Epstein–Barr virus, have enabled the identification of populations most likely to benefit from immunotherapy and targeted therapies. However, a low incidence of MSI-high in metastatic disease (3%) has been reported for a recent cohort of patients with stage IV disease [120]. Therefore, it is still challenging to treat cancers for which markers for the determination of effective immunotherapy are lacking.

Galectin-9 has also been reported to be associated with gastric cancer. Immunohistochemical analysis showed that 86.2% of tumor tissues from gastric cancer patients were positive for galectin-9 and 60.0% for TIM-3. Both high expression of galectin-9 and low expression of TIM-3 were significantly associated with longer overall survival in gastric cancer patients [121] (Table 1). In this report, differentiation between extracellular and intracellular galectin-9 expression was complicated because it was performed by immunohistochemistry in human gastric cancer tissues. On the other hand, TIM-3 positive cells may decrease due to apoptosis induced by increased extracellular galectin-9. In another study, the galectin-9 positive group in gastric cancer tissues had significantly lower all-cause and gastric cancer-specific mortality rates than the galectin-9 negative group [122]. Galectin-9 inhibits the proliferation of gastric cancer cell lines by altering miRNAs in vitro, mainly by inducing apoptosis [96]. It has been reported that chemoradiotherapy increases the expression of galectin-9 and PD-L1 on the cell membrane in gastric cancer cell lines [123], and combination therapy in actual clinical practice is expected in the future.

## 6. Colorectal Cancer and Galectin-9 Apoptosis

Colorectal cancer has the third-highest incidence rate and the second-highest mortality rate. Colorectal cancer accounts for about one in ten cancer patients [124]. Despite remarkable progress in the treatment of early-stage colorectal cancer using endoscopy and laparoscopy [125], there is no effective treatment for patients with advanced colorectal cancer. Treatment of advanced colorectal cancer is based on chemotherapy, radiation therapy, and immunotherapy [126] (Table 1). Due to medical advances, significant efforts have been made to characterize various prognostic factors and biomarkers to clarify the indications, regimen type, dosage, and duration of drug therapy in patients with colorectal cancer [127].

Galectin-9 is closely linked to colorectal cancer. Galectin-9 expression is downregulated in colon tumor tissues [101], and high expression of galectin-9 is associated with improved overall survival in colorectal cancer [101,128]. Furthermore, galectin-9 is involved in natural killer (NK) cell chemotaxis by regulating the polarity of F-actin in NK cells [128]. In a mouse model using colon cancer cells, intravenous administration of galectin-9 reduced the number of metastases in the lung, suggesting that secreted Gal-9 suppresses metastasis [129]. In addition, galectin-9 inhibits cell proliferation of colorectal cancer cell lines in vitro and in vivo, and its mechanism is reported to be the induction of apoptosis via miRNA alteration [130]. Notably, miR-455-5p promotes tumor growth by targeting galectin-9 and inhibiting apoptosis in colon cancer [131]. Thus, galectin-9 may be a new target for immunotherapy of colorectal cancer.

## 7. Hepatocellular Carcinoma and Galectin-9 Apoptosis

In HCCs, galectin-9 can potentially regulate tumor progression directly and indirectly. In the direct pathway, cell surface galectin-9 or galectin-9 administered into the culture medium inhibits cell proliferation of HCC. In the indirect mechanism, galectin-9 controls tumor immunity against HCC. In a previous analysis, enhanced tumor expression of galectin-9 correlated with a better prognosis of patients with HCC [132] (Table 1). Circulating galectin-9 indicates a better prognosis [111].

It has been reported that galectin-9 induces apoptosis in HCC cell lines in vitro [18]. The mechanism of apoptosis in hepatocellular carcinoma is now known to involve an intrinsic pathway involving endoplasmic reticulum stress rather than a death receptor pathway via activation of caspase-12 and caspase-8 and upregulation of Chp and Bip [133,134]. Since the representative galectin-9 receptor, T-cell immunoglobulin and mucin-domain containing-3 (TIM-3), is not expressed on the surface of HCC cell lines, and galectin-9-induced apoptosis is inhibited by lactose administration, this receptor should be glycosylated by β-galactoside [18]. In addition, interferon induces galectin-9 expression in HCC cells [104]. The background for the enhancement of galectin-9 expression in HCC cells by interferon is that galectin-9 is a target of microRNA 22 (miR-22), and restoring galectin-9 expression in miR-22 overexpressing cells enhanced its antitumor effect [104].

Galectin-9 expression was examined in the tissue samples of 200 HCC patients by immunohistochemistry and correlated with the histopathologic grade, lymph node metastasis, vascular invasion, and intrahepatic metastasis (*p* < 0.05). In addition, the survival time in patients with galectin-9 expression was statistically longer than those with negative lesions as assessed by the Log-rank test (*p* < 0.0001) [102]. In another study, lack of, or low, tumor expression of galectin-9 (*p* < 0.001) was involved in poor HCC-specific survival, independent from baseline clinicopathologic characteristics [132]. Using 90 surgically resected specimens, we measured galectin-9 mRNA expression in hepatocarcinoma and adjacent cancer tissue and found significant differences in terms of pathologic differentiation, TNM, and recurrent metastasis (*p* < 0.05) [135].

Furthermore, in a meta-analysis based on 43 cohorts and 33 studies, high tissue galectin-9 expression was significantly associated with longer overall survival (HR = 0.56, 95% CI = 0.44–0.71, *p* < 0.001) and vascular invasion (OR = 0.60, 95% CI = 0.37–0.97, *p* = 0.037) was significantly associated with improved overall survival [136]. Similarly, in another meta-analysis based on 11 studies, including 1957 patients, a pooled HR of high galectin-9 expression was correlated with improved overall survival [137]. However, there have also been reports that galectin-9 expression in hepatocellular carcinoma tissue did not directly correlate with patient outcomes. Galectin-9 expression in primary tumors was not significantly higher in tumor tissue compared to adjacent non-tumor tissue (*p* = 0.222) [138].

When restricted to patients with hepatitis B virus (HBV)-associated HCC, galectin-9 expression-positive individuals were associated with lymph node metastasis (*p* = 0.029), Ki-67 proliferation index (*p* = 0.009), and poor prognosis [106], indicating that galectin-9 expression can be used as an independent prognostic marker for HBV-associated HCC. In the same patient group, the group with a high expression of TIM-3 on CD4+ T cells had shorter overall survival than the group with a low expression of TIM-3. Since extracellular galectin-9 can bind to TIM-3, galectin-9 may inhibit liver tumor cell immunity [12].

Furthermore, it has been suggested that the NK cell population, which constitutes an important antitumor effector in the liver, may also be a target of galectin-9 in immunosuppression. It has been reported that ligation of galectin-9 to NK cells decreases IFN-γ production independently of TIM-3. In other words, galectin-9 suppresses immune activating genes, including eight genes involved in the NK cell-mediated cytotoxic pathway, which suppresses lymphocyte activity and reduces IFN-γ production [139].

## 8. Cholangiocarcinoma (CCA) and Galectin-9 Apoptosis

Cholangiocarcinoma (CCA) is a malignant tumor in the biliary system, a bile drainage tract, and is dominated by cancer types originating from the biliary epithelium. Furthermore, cholangiocellular carcinoma is an epithelium malignancy consisting of cells resembling or derived from the bile duct epithelium. It originates from within the liver and is the second most common primary liver malignancy after HCC, accounting for approximately 15% of primary liver cancers and 3% of gastrointestinal malignancies [140,141]. CCA has shown an increasing trend in recent decades, with an incidence rate of 0.3–6 per 100,000 people per year worldwide [140]. In some Asian countries such as Thailand and Cambodia, the incidence rate can be as high as 85 per 100,000 people due to liver leech infection [141]. The lack of specific clinical symptoms in the early stages of CCA development makes diagnosis difficult, and once the disease progresses to intermediate and advanced stages, treatment options are limited [142]. In recent years, treatment of CCA has diversified, and more mature and effective surgical, radiotherapeutic, and chemotherapeutic modalities are being used as first-line treatment, but even patients who have undergone radical treatment have a poor prognosis, with a reported five-year survival rate of 20–35% [143,144]. The development of novel therapies targeting cancer immune mechanisms, including galectin-9, may be necessary. 

There is no report of the antitumor effect of galectin-9 on CCA other than in our previous study, where we showed that galectin-9 had antitumor effects on CCA cell lines in vitro and in vivo [19] (Table 1). Galectin-9 treatment of CCA cells showed antitumor effects by accumulating cleavage of cytokeratin 18 (CCK18), which occurs as an early event in apoptosis following effector caspases. We also found that CCA cells treated with galectin-9 increased the expression of cytochrome c released from apoptosis-damaged mitochondria and contributed to the activation of caspase-9. On the contrary, the expression levels of cell cycle-related proteins (cyclin D1, cyclin E, cdk4, cdk6, and cdk2) were not altered after galectin-9 treatment, and cell cycle arrest was not observed. Furthermore, galectin-9 decreased the expression of hepatocyte growth factor receptor (HGFR), fibroblast growth factor receptor 3 (FGFR3), phosphorylated epidermal growth factor receptor (p-EGFR), and phosphorylated epidermal growth factor-1 receptor (p-IGF-1R) in CCA cells, and changes in the activity of these receptor tyrosine kinases (RTKs) might have contributed to the antitumor effect.

## 9. Pancreatic Cancer and Galectin-9 Apoptosis

Pancreatic cancer is the seventh leading cause of cancer-related death in both males and females, with 55,000 males and 44,000 females dying of pancreatic cancer worldwide in 2020 [84]. The most common malignant neoplasm of the pancreas is pancreatic ductal adenocarcinoma (PDAC), and the term pancreatic cancer generally refers to this entity. The five-year survival rate for patients with PDAC was reported to be only 9%. The incidence of PDAC is on the rise and is predicted to become the second leading cause of cancer deaths by 2030 [145]. The main reasons for the increased morbidity and mortality of PDAC are a low early detection rate, high surgical mortality rate, low sensitivity to radiation chemotherapy, and high rate of recurrence and metastasis [146]. Since conventional systemic chemotherapy has limited therapeutic efficiency against PDAC, understanding the cancer immune microenvironment, including galectin-9 in PDAC, and exploring its potential as a new therapeutic strategy may improve the prognosis of this deadly disease (Table 1).

Evidence suggests that changes in galectin expression in PDAC tissues may be involved in tumorigenesis, proliferation, progression, angiogenesis, metastasis, and immune responses [147,148]. It was suggested that high expression of several galectins, including galectin-9, was a predictor of poor prognosis in a recent meta-analysis aimed at clarifying the accurate diagnostic and prognostic role of galectins in PDAC [149]. Another study reported that γδT cells in the blood of PDAC patients showed higher expression of galectin-9 than γδT cells in healthy controls and that galectin-9 serum levels are a useful biomarker for detecting PDAC [112,150]. Galectin-9 polarized macrophages to the primitive M2 phenotype and suppressed cytokine secretion by T cells.

Our study showed that galectin-9 inhibited cell proliferation and tumor growth of PDAC cells in vitro [20]. In human PDAC cell lines, as in other gastrointestinal cancer cell lines, galectin-9 increased the expression of CCK18 and cytochrome c. These results suggest that galectin-9 may induce apoptosis in PDAC cells through intrinsic apoptotic effects in caspase-dependent and caspase-independent pathways. Although the expression of cyclin E and cdk4 were decreased after galectin-9 treatment, flow cytometry revealed that galectin-9 did not occur during G0/G1 arrest of PDAC cells in vitro and may not be related to changes in various cell cycle-related proteins. In addition, galectin-9 decreased the expression of p-EGFR and phosphorylated tyrosine kinase with lg and EGF homology domains 2 (Tie-2), tropomyosin receptor kinase C (TrKC), muscle-associated receptor tyrosine kinase (MuSK), anaplastic lymphoma kinase (ALK), ephrin type-A receptor 10 (EphA10), and related tyrosine kinase orphan receptor (RYK) according to RTK array analysis. It is known that the EGFR family and its ligands are frequently overexpressed in PDAC cells [151] and correlate with patient prognosis [152]. The antitumor effect of galectin-9 on PDAC cells may be dependent on the change in the expression of certain proteins from the EGFR family. In another study using metastatic liver cancer cell lines derived from pancreatic cancer, including KMP2, KMP7, and KMP8, we demonstrated the in vitro antitumor effect of galectin-9 with apoptosis as the main mechanism [107]. Galectin-9 increased the level of CCK18 and enhanced the expression of apoptosis-related proteins, including caspase-7, cleaved caspase-3, cleaved poly ADP-ribose polymerase (PARP), cytochrome c, Second mitochondria-derived activator of caspase (Smac)/direct inhibitor of apoptosis-binding protein with low pl (Diablo), and high-temperature requirement protein A2 (HTrA2). However, galectin-9 did not affect the expression of various cell cycle-related proteins in metastatic liver cancer and PDAC cells. Therefore, these data suggest that galectin-9 may be an effective therapeutic agent against PDAC or its liver metastasis.

Recent reports have demonstrated that an exosome-based dual-delivery biosystem enhances PDAC immunotherapy and restores tumor immunosuppression in M2-like tumor-associated macrophages by disrupting the galectin-9/dectin-1 axis [153]. This delivery system was constructed by injecting the bone marrow mesenchymal stem cell exosome and galectin-9 small interfering RNA by the electroporation method and surface modification with oxaliplatin producing an immunogenic cell death trigger. The combination therapy of galectin-9 and oxaliplatin has the potential to induce antitumor immunity through polarization of tumor suppressive macrophages, mobilization of cytotoxic T lymphocytes, and down-regulation of regulatory T cells to achieve a significant therapeutic effect in PDAC treatment.

## 10. Conclusions

Conventional molecular-targeted drugs that target molecules involved in signaling pathways that inhibit tumor growth, including apoptosis, face the major problem of drug resistance. The binding of glycans to receptor proteins and various glycan-mediated pathways, which involve completely different mechanisms, have recently attracted great attention and may be new targets for drugs that efficiently control apoptosis and tumor growth, thereby inhibiting cancer cell proliferation. In particular, galectin-9 has been shown to be involved in the dysregulation of apoptotic mechanisms in oncogenesis and directly induces apoptosis in gastrointestinal cancer cells. However, the exact pharmacokinetics of galectin-9 in humans and the cell surface receptors involved in the induction of cancer apoptosis by galectin-9 have not been identified in previous studies. Moreover, the intracellular pathways involved in apoptosis have not been fully investigated. Elucidation of the detailed mechanisms involved in galectin-9 induction of apoptosis in gastrointestinal cancers is expected to be a potential new therapeutic approach for the treatment of gastrointestinal cancers.

## Figures and Tables

**Table 1:** Function of galectin-9 in gastroenterological cancers.

Cancer Type	Function and Clinical Significance	References
Esophageal cancer	Prognosis	[17,88]
Gastric cancer	Survival	[96,97,98]
Colon cancer	Proliferation, antitumor immunity	[99,100,101]
Liver cancer	Cell adhesion	[102,103]
	Invasion	[104,105]
	Metastasis	[104,106,107]
	Apoptosis	[18,105,107]
	Immunosuppression	[104,106,108]
	Progression	[104,105,109,110]
	Prognosis and survival	[106,111]
Cholangiocarcinoma	Proliferation	[19]
Pancreatic cancer	Apoptosis, proliferation, and growth	[20,107]
	Antitumor immunity	[112,113,114,115]

## Data Availability

Not applicable.
